# Integrated Yoga and Naturopathy Interventions to Modify Functional Disability in Patients With Spinal Cord Injury: A Randomized Controlled Trial

**DOI:** 10.7759/cureus.57686

**Published:** 2024-04-05

**Authors:** Sujatha KJ, Manjunath N K, Prashanth Shetty

**Affiliations:** 1 Natural Therapeutics, Sri Dharmasthala Manjunatheshwara (SDM) College of Naturopathy and Yogic Sciences, Mangalore, IND; 2 Yoga, Swami Vivekananda Yoga Anusandhana Samsthana (SVYASA), Bangalore, IND; 3 Yoga, Sri Dharmasthala Manjunatheshwara (SDM) College of Naturopathy and Yogic Sciences, Mangalore, IND

**Keywords:** physical rehabilitation, yoga, naturopathy, functional disability, spinal cord injury

## Abstract

Objective: This study aimed to evaluate the use of integrated yoga and naturopathy intervention to modify functional disability and improve independence in patients with spinal cord injury.

Materials and methods: In this randomized controlled trial, 48 spinal cord injury patients receiving residential rehabilitation, aged between 23 and 57 years (37.9±11.8) of both genders, were randomly allocated to two groups: (i) experimental group (naturopathy and yoga) and (ii) control group (waitlist with routine care). Subjects were assessed on day 1 (baseline), day 30 (intense phase), and day 90 (follow-up). Assessments were done using the Spinal Cord Independence Measure (SCIM), handheld myometry (HHM), time up and go (TUG), Berg Balance Scale (BBS), and 10-meter walk test (10MWT).

Results: There were no significant differences at baseline between groups for all the variables (*p*>0.05) through one-way analysis of variance (ANOVA). Repeated measures ANOVAs (RM-ANOVAs) were performed to compare between assessments and the groups (*p*<0.05). Post hoc shows that there is significant SCIM (*p*<0.001), HHM (*p*<0.001), TUG (*p*<0.001), BBS (*p*<0.001), and 10MWT (*p*<0.001).

Conclusion: The present study shows that there is significant improvement in the functions of both yoga and naturopathy and the control group. So, yoga and naturopathy can be considered as adjuvant along with routine care of physical therapy in spinal cord injury rehabilitation programs.

## Introduction

Functional disability often ensues from spinal cord injury (SCI). The impairment is visible in the ability to execute activities of daily living (ADL). The assessment of ADLs or functional disabilities considers limitations in activities including walking, climbing stairs, grooming, going to the toilet, getting out of bed, and shifting into a chair [1]. Functional impairments with activity restrictions and limitations related to mobility as well as self-care can result in difficulty in pursuing employment, sustaining social relationships, participating in leisure activities, and engaging as a member of the community [2]. The degree of motor function, age of the patient, length of therapy, existence of pressure sores or spasticity, site of the injury, and severity of the damage are all associated with functional disability in SCI [3]. Globally, 2-5 lakh people suffer from SCI, with a male-to-female ratio of 2:1 among adults. Up to 90% of these cases are due to traumatic causes, though the proportion of nontraumatic spinal cord injuries appears to be growing [4]. According to the Rehabilitation Council of India, 15,000 cases of SCI are reported on average every year, with 0.15 million cases occurring annually. India is among the developing nations where SCI is becoming more common, and the associated healthcare burden is predicted to be comparable to that of the developed world [5]. Complementary and integrative medicine are growing in popularity in the United States, reducing the healthcare burden. In 2012, 33.2% of adult US citizens said they had used alternative therapy at some point in the past year. The most common practices were deep breathing exercises, yoga, tai chi, and chiropractic adjustments; nevertheless, the use of qi gong, yoga, and dietary supplements increased linearly between 2002 and 2012. The reported usage of complementary therapies varies widely among individuals with SCI, ranging from 14% to 73%. Studies that focused on individuals with both SCI and chronic pain showed higher rates. However, most of the samples have been tiny, and prior research has concentrated on the consequences of SCI as well as the initial weeks of inpatient rehabilitation for patients who have recently received a diagnosis of SCI and who have been informed about its potential impact on physical or psychological outcomes [6].

This study is designed to assess the use of integrated yoga and naturopathy intervention to modify functional disability and improve independence in patients with SCI.

## Materials and methods

Ethical consideration

The study protocol was approved by the Institutional Ethics Committee of Swami Vivekananda Yoga Anusandhana Samsthana (approval number: RES/IEC-SVYASA/202/2021) and was registered in the Clinical Trials Registry-India (CTRI/2021/10/037130).

Participants

The sample size was calculated using the G*Power software by fixing the alpha at 0.05, the power at 0.8, and the effect size at 0.92 based on the mean and standard deviation of low frequency (%) from a previous study [7]; considering 20% attrition rate, the optimal sample size was 24 in each group. The subjects were recruited from the spinal rehabilitation center as per the diagnostic criteria of the American Spinal Injury Association (ASIA) scoring system [8]. Recruits included 48 subjects of both genders (males: 95.64%, females: 4.16%), ranging in age from 37.9±11.8 years to 7 years, with varying injury durations while hospitalized for rehabilitation. The inclusion criteria were the ability to engage in mild physical activity, participate in the intervention safely, and receive SCI grades between B and D in the ASIA scoring system. The study was explained to the participants, whose signed informed consent was taken. Institutional ethics committee approval was obtained.

Data

The design of the study was a randomized controlled trial. Subjects were recruited on a prospective basis and were randomly allocated to two groups by using computer-generated randomization: (i) the experimental group and (ii) the control group. The subjects of the experimental group received an integrated yoga and naturopathy intervention as an adjuvant therapy to routine medical care including physiotherapy for three months as prescribed by the consulting physician, whereas the control intervention group received only routine medical care with physiotherapy for three months. Subjects from both groups were assessed on day 1, day 30, and day 90. The intervention was provided in two phases: intense treatment (30 days) and follow-up (60 days). The time of day was kept constant for both sessions; individual sessions were 22:30 minutes in duration.

Assessments were done by using a 10-meter walk test (10MWT), handheld myometry (HHM), time up and go (TUG), Berg Balance Scale (BBS), and the Spinal Cord Independence Measure (SCIM).

Intervention

Intervention was provided in two phases: the intense treatment phase (30 days) and the follow-up phase (60 days). The assessments were done on the first, 30th, and 90th days. The wait-list control group received the same treatments after three months. The subjects received naturopathy intervention such as a cold and revulsive (three minutes hot and one minute cold) spinal compress for 30 minutes of five rounds along with yogic intervention [9] according to the protocol shown in Table 1.

**Table 1:** Yoga protocol for 90 days Reference: [9] DRT: deep relaxation technique

Type of practice	Name of the practice	Duration (frequency) practice
Loosening exercises (Sukshma Vyayama) of the upper limb	Finger movements	Five minutes (five rounds for each movement)
	Wrist movements	
	Elbow movements	
	Shoulder movements	
Loosening exercises (Sukshma Vyayama) of the lower limb	Toe movements	Five minutes (five rounds for each movement)
	Ankle movements	
	Knee movements	
	Hip movements	
Asanas (with support or props)	Utkatasana	Two minutes (two repetitions)
	Ardha Chakrasana	
	Ardhakati Chakrasana	
	Vakrasana	
Pranayamas	Vibhagiya Pranayama (sectional breathing)	Six rounds (three minutes)
	Nadi Shuddhi Pranayama	Nine rounds (five minutes)
	Bhramari Pranayama	Nine rounds (five minutes)
Relaxation	DRT	10 minutes

Both groups underwent physiotherapy intervention as routine care. The physiotherapy exercises were (1) proprioceptive neuromuscular facilitation, (2) slow and sustained stretching, (3) strengthening of antigravity muscles, (4) functional electrical stimulation, and (5) gait training. All these lasted for 60 minutes, six days a week for one month. Later, the participants were asked to follow up for the next 60 days.

Statistical analysis

Data were represented in mean±standard deviation. Repeated measures analysis of variance (RM-ANOVA) followed by post hoc Bonferroni's correction was performed for all the variables, with the level of significance at p<0.05. Data were analyzed using Jamovi 2.4.8.

## Results

**Figure 1:**
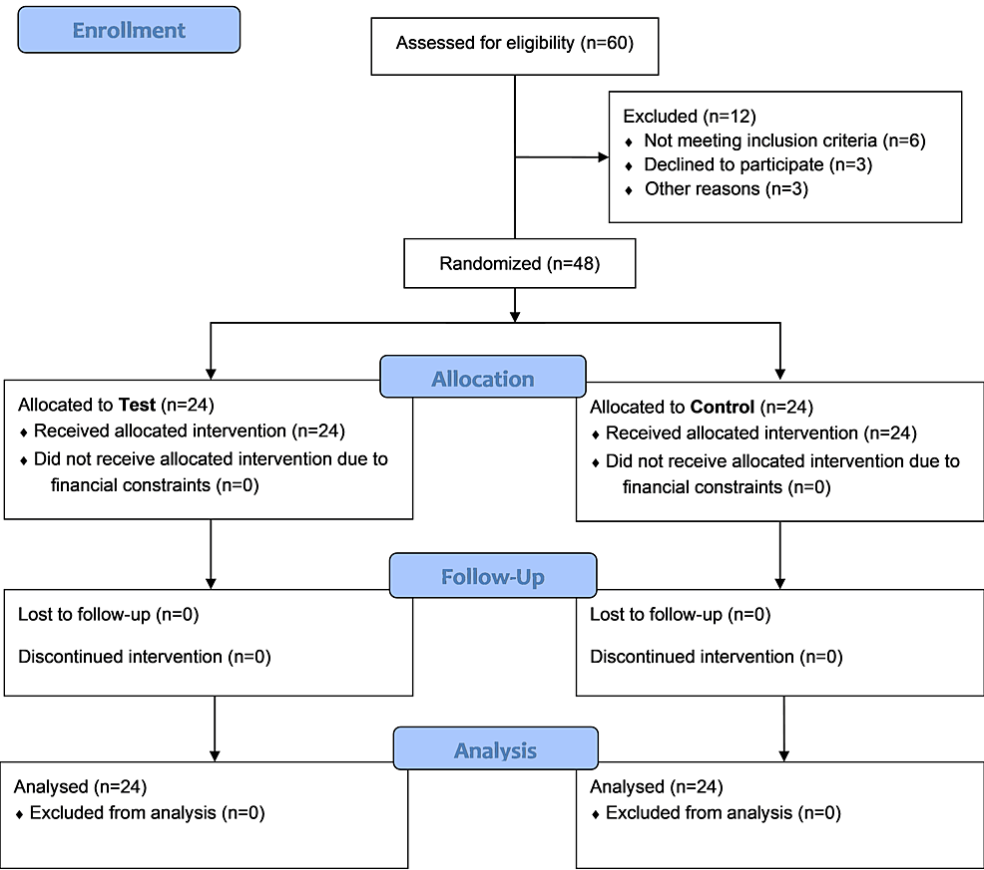
Illustration of study plan

Initially, 60 patients in total were contacted. The study participants' flowchart is shown in Figure 1.

A total of 48 patients who agreed to participate fulfilled the requirements for inclusion, provided written informed consent, and were assigned to either the yoga and naturopathy group (n=24) or the control group (n=24).

The baseline values of both the groups for all the variables were compared using separate one-way ANOVAs, and the results suggested that there were no significant differences at baseline between the groups (p>0.05).

RM-ANOVAs were performed to compare the differences among the assessments (baseline, first month, and three months or baseline and three months as within-subject factors) and between the two groups. We also performed a post hoc subgroup analysis to compare results at baseline and then after the first month and after three months.

SCIM

Self-Care

An RM-ANOVA was done on the self-care score in the SCIM. Results showed significant group-by-time interaction effects (F=65.5, MS=29.88, p<0.001) and between-subject effects (F=6.21, MS=28.44, p<0.016). However, there was a significant within-subject difference (F=338.1, MS=154.15, p<0.001). Post hoc tests using Bonferroni's correction showed significant differences between the yoga and control groups in post1 and post2 after yoga (p<0.05) as shown in Table 2.

**Table 2:** SCIM score recorded on days 1, 30, and 90 in both test and control **: p<0.01, ***: p<0.001, †: p<0.05, † † †: p<0.001, repeated measures ANOVA with post hoc analysis *: comparing the day 1 values with respective day 30 and day 90 values; †: comparing day 30 and day 90 values RSM: respiratory and sphincter management; SCIM: Spinal Cord Independence Measure; ANOVA: analysis of variance

Variable	Test			Control		
	Day 1	Day 30	Day 90	Day 1	Day 30	Day 90
Self-care	8.25±1.36	10.5±1.22***	13.4±1.69***^,^ † † †	8.71±1.20	9.96±1.23***	10.8±1.36***^,^ † † †
RSM	34.7±1.2	35.8±1.17***	36.6±0.83***^,^ † † †	34.8±1.38	35.5±1.32***	35.9±1.14***^,^ † † †
Mobility	21.2±2.57	24.6±2.52***	28.1±2.34***^,^ † † †	21.3±2.58	22.7±2.54***	23.9±2.52***^,^ † † †

Respiratory and Sphincter Management (RSM)

An RM-ANOVA was done on the RSM score in the SCIM. Results showed significant group-by-time interaction effects (F=3.17, MS=1.896, p<0.047) but not between-subject effects (F=0.983, MS=3.06, p<0.32.7). However, there was a significant within-subject difference (F=47.24, MS=28.25, p<0.001). Post hoc tests using Bonferroni's correction showed significant differences between the yoga and control groups in post1 and post2 after yoga (p<0.05) as shown in Table 2.

Mobility

An RM-ANOVA was done on the mobility score in the SCIM. Results showed significant group-by-time interaction effects (F=82.5, MS=55.39, p<0.001), between-subject effects (F=8.17, MS=144, p<0.006), and a within-subject difference (F=399.9, MS=268.39, p<0.001). Post hoc tests using Bonferroni's correction showed significant differences between the yoga and control groups in post1 and post2 after yoga (p<0.05) as shown in Table 2.

Handgrip

An RM-ANOVA was performed on the handgrip score in SCIM. On the right hand, results showed significant group-by-time interaction effects (F=3.3, MS=3.4, p<0.04), between-subject effects (F=4.24, MS=297.6, p<0.04], and a significant within-subject difference (F=122.52, MS=126.22, p<0.001). On the left hand, results showed significant group-by-time interaction effects (F=4.47, MS=2.5, p<0.01), nonsignificant between-subject effects (F=4.47, MS=2.5, p<0.3), and a significant within-subject difference (F=291.5, MS=163.3, p<0.001). Post hoc tests using Bonferroni's correction showed significant differences between the yoga and control groups in post1 and post2 after yoga (p<0.05) as shown in Table 3.

**Table 3:** Handgrip score recorded on days 1, 30, and 90 in both test and control **: p<0.01; ***: p<0.001; †: p<0.05; † † †: p<0.001, repeated measures ANOVA with post hoc analysis *: comparing the day 1 values with respective day 30 and day 90 values; †: comparing day 30 and day 90 values ANOVA: analysis of variance

Variables	Test			Control		
	Day 1	Day 30	Day 90	Day 1	Day 30	Day 90
Right hand (N)	18.2±5.75	19.9±4.74***	21.9±4.31***^,^ † † †	15.9±5.08	16.9±4.95***	18.6±4.31***^,^ † † †
Left hand (N)	19±5.94	21.2±5.55***	23.1±4.74***^,^ † † †	18.1±4.74	19.4±4.47***	21.4±4.03***^,^ † † †

TUG

An RM-ANOVA was performed on the TUG score in the SCIM. Results showed significant group-by-time interaction effects (F=68.1, MS=322.9, p<0.001), nonsignificant between-subject effects (F=1.19, MS=895, p<0.28), and a significant within-subject difference (F=68.1, MS=322.9, p<0.001). Post hoc tests using Bonferroni's correction showed significant differences between the yoga and control groups in post1 and post2 after yoga (p<0.05) as shown in Table 4.

**Table 4:** TUG, 10MWT, and BBS scores recorded on days 1, 30, and 90 in both test and control **: p<0.01; ***: p<0.001; †: p<0.05; † † †: p<0.001, repeated measures ANOVA with post hoc analysis *: comparing the day 1 values with respective day 30 and day 90 values; †: comparing day 30 and day 90 values TUG: time up and go; 10MWT: 10-meter walk test; BBS: Berg Balance Scale; ANOVA: analysis of variance

Variables	Test			Control		
	Day 1	Day 30	Day 90	Day 1	Day 30	Day 90
TUG (s)	87.5±16.7	78.4±15.3***	66.5±14.4***^,^† † †	88±16.8	82.3±16.3***	77.1±15.8***^,^† † †
10MWT (m/s)	0.0779±0.0118	0.0856±0.0140***	0.0979±0.0166***^,^† † †	0.0801±0.0169	0.0872±0.0197***	0.0930±0.0265***^,^† † †
BBS	33.8±4.49	37.2±3.27***	40.7±1.99***,† † †	33.6±4.54	35.0±4.15***	36.5±3.67***,† † †

10MWT

An RM-ANOVA was done on the BBS score in SCIM. Results showed significant group-by-time interaction effects (F=21.6, MS=1.87, p<0.001), nonsignificant between-subject effects (F=0.006, MS=5.87, p<0.93), and a significant within-subject difference (F=376.5, MS=0.003, p<0.001). Post hoc tests using Bonferroni's correction showed significant differences between the yoga and control groups in post1 and post2 after yoga (p<0.05) as shown in Table 4.

BBS

An RM-ANOVA was done on the BBS score in the SCIM. Results showed significant group-by-time interaction effects (F=39.5, MS=49.01, p<0.001), between-subject effects (F=4.11, MS=166.8, p<0.04), and a significant within-subject difference (F=235.7, MS=296.2, p<0.001). Post hoc tests using Bonferroni's correction showed significant differences between the yoga and control groups in post1 and post2 after yoga (p<0.05) as shown in Table 4.

## Discussion

The results of this trial indicated that modified autonomic dysfunction and improvement in functional outcomes were seen more often in the individuals with SCI who participated in structured yoga and naturopathy interventions with physical rehabilitation compared to physical rehabilitation alone. The results are concurrent with previous mind-body interventions like yogasana, pranayama, and meditation that have helped reduce pain and enhance functional independence and quality of life in SCI patients [9,10]. Although there was a significant improvement in both groups, the group that practiced yoga and naturopathy demonstrated a greater magnitude of change (mean difference) than the group that only received physical rehabilitation. The goal of the current study was to close the knowledge gap regarding potential mechanisms to enhance the functional outcomes of SCI. In addition to yoga, the subjects received naturopathy, which modifies autonomic dysfunction and helps in the control of vascular physiology [11], thermoregulation [12], splanchnic outflow, and adrenaline outflow in SCI patients [13]. Naturopathy interventions like revulsive compresses, when used in conjunction with other interventions, have been shown to improve functional outcomes and reduce pain. These findings are in line with earlier research on cervical spondylosis by Sujatha and Manjunath [14].

In the present study, the improvement in functional outcomes, pain, and quality of life could be due to a profound modification of autonomic dysfunction and a reduction of pain that leads to better functional independence. The ANS's supraspinal influence is disturbed following an SCI, which results in parasympathetic dominance and sympathetic blunting. This causes cardiac dysrhythmias, systemic hypotension, bronchoconstriction, copious respiratory secretions, and uncontrollably high blood pressure, bowel, and bladder functions [15]. The results of the present study are encouraging, in that the yoga and naturopathy interventions along with physical rehabilitation helped improve functional independence. The conclusive evidence derived from the long-term follow-up of three months reinforces previous studies using a short-term follow-up [16-18].

One strength of the present study was that all the participants stayed in the same rehabilitation center for a period of three months, which helped the subjects adhere to the interventions.

Future trials could focus more on early interventions for individuals suffering from SCI, which may result in better and earlier improvement in autonomic dysfunction, functional outcomes, and quality of life.

## Conclusions

The present study shows that there was a significant improvement in functional outcomes in both the yoga and naturopathy group and the control group. So, we can include yoga and naturopathy function as adjuvant alongside routine care of physical therapy in a SCI rehabilitation program.
